# Investigation of Gas Sensing Performance of CuO/Cu_2_O Thin Films as a Function of Au-NP Size for CO, CO_2_, and Hydrocarbons Mixtures

**DOI:** 10.3390/nano15100705

**Published:** 2025-05-08

**Authors:** Christian Maier, Larissa Egger, Anton Köck, Sören Becker, Jan Steffen Niehaus, Klaus Reichmann

**Affiliations:** 1 Materials Center Leoben Forschung GmbH, Roseggerstraße 12, 8700 Leoben, Austria; larissa.egger@mcl.at; 2 Institute for Chemistry and Technology of Materials, Graz University of Technology, Stremayrgasse 9, 8010 Graz, Austria; k.reichmann@tugraz.at; 3 Fraunhofer Center for Applied Nanotechnology, Grindelallee 117, 20146 Hamburg, Germany; soeren.becker@iap.fraunhofer.de (S.B.); jan.steffen.niehaus@iap.fraunhofer.de (J.S.N.)

**Keywords:** metal oxide, CuO, Cu_2_O, Au nanoparticles, drop coating, thermal evaporation, spray pyrolysis, gas sensors

## Abstract

This study examines the impact of Au nanoparticles (Au-NPs) on the chemoresistive gas sensing properties as a function of particle size. The sensing material is composed of ultrathin CuO/Cu_2_O films, which are fabricated by either thermal deposition technology or spray pyrolysis. These are used on a silicon nitride (Si_3_N_4_) micro hotplate (µh) chip with Pt electrodes and heaters. The gas sensing material is then functionalised with Au-NP of varying sizes (12, 20, and 40 nm, checked by transmission electron microscopy) using drop coating technology. The finalised sensors are tested by measuring the electrical resistance against various target gases, including carbon monoxide (CO), carbon dioxide (CO_2_), and a mixture of hydrocarbons (HC_Mix_), in order to evaluate any cross-sensitivity issues. While the sensor response is markedly contingent on the structural surface, our findings indicate that the dimensions of the Au-NPs exert a discernible influence on the sensor’s behaviour in response to varying target gases. The 50 nm thermally evaporated CuO/Cu_2_O layers exhibited the highest sensor response of 78% against 2000 ppm CO_2_. In order to gain further insight into the surface of the sensors, a scanning electron microscope (SEM) was employed, and to gain information about the composition, Raman spectroscopy was also utilised.

## 1. Introduction

In the 1950s, Brattain and Bardeen demonstrated the dependence of the resistance of germanium (Ge) on the atmosphere, which marked the birth of electrical gas sensing. Subsequently, in the 1970s, Taguchi developed the first semiconducting metal oxide gas sensor (SMOX), which was based on SnO_2_ as the sensitive layer [1]. In light of the growing prevalence of progressive health aspects and the enactment of governmental regulations, it is imperative to assess the levels of air pollution both indoors and outdoors. In particular, the presence of elevated concentrations of gases such as CO or CO_2_ in domestic environments can give rise to adverse health effects and may result in respiratory problems [2]. The gas sensor most commonly employed for the detection of carbon dioxide is the non-dispersive infrared (NDIR) sensor, which facilitates a wide detection range [3]. However, these sensors are characterised by substantial dimensions, thereby precluding miniaturisation.

In the present era, a significant proportion of research is directed towards the development of SMOX gas sensors, which are characterized by their low production costs, extensive range of detectable gases, and diverse application areas [4].

CuO, when combined with other materials, has been identified as a promising material candidate due to its sensitivity to a wide range of target gases, including volatile organic compounds (VOCs) [5], hydrogen sulphide (H_2_S) [6], and nitrogen dioxide (NO_2_) [7].

Duan et al. [8] reported that LaFeO_3_ sensors doped with cobalt (Co) exhibited a sensor response of less than 8% when exposed to 10,000 ppm of CO_2_. Furthermore, the potential of CuO in the detection of CO_2_ is a prevalent area of research. A summary of CuO and material combinations for the detection of CO_2_ is provided in the literature [9]. Different deposition techniques, such as magnetron sputtering [10], thermal oxidation [11], spray pyrolysis [12], sol-gel [13], and hydrothermal [14], are established for the production of sensitive CuO layers. The necessity of an adhesion layer, and its significant influence on sensor performance towards different target gases, is contingent on the fabrication method, substrate, and thickness of the sensitive layer [15].

However, there are still some other issues to be resolved, such as integration problems [16] with complementary metal oxide semiconductor (CMOS) technology, selectivity, and cross-sensitivity. In order to achieve a high-efficiency gas sensor (low production costs, low power consumption, and miniaturization), it is necessary to incorporate additives such as noble metals, which result in an increase in sensitivity, selectivity, and a reduction in operating temperature [17]. The noble metal exhibits a markedly higher electrical conductivity, which alters the conduction channel of the metal oxide, thereby enhancing the sensing performance [18].

In general, two possible mechanisms are identified for electrical and chemical sensitization that enables gas sensing with doped metal oxides [19]. The role of noble metals such as platinum (Pt) in chemical sensitization is the activation and spillover of the target gas. Here, a change in the adsorbed oxygen is the gas-sensing parameter. In contrast, in electronic sensitization, noble metals such as palladium (Pd) interact with the target gas and act as an electron donor or acceptor to the metal oxide [20].

SnO_2_ is the most extensively studied material as a sensing layer for n-type semiconducting metal oxides, with many doping variations such as mono-, bi-, and trimetallic NPs [21]. Korotcenkov et al. [22] demonstrated that the diameter of Au-NP has a significant influence on the conductivity and sensor response to CO, hydrogen (H_2_), and ozone (O_3_). This paper also assumes that gold does not alter the chemical state and that the process responsible for enhancing the sensor response occurs in the vicinity of the Au-NPs. In [23], the highest sensor response recorded was 356% towards 2000 ppm CO_2_ at 50% relative humidity (r.h.) for the combination of the p-type CuO, which had been functionalised by drop coating technique with citrate-stabilised Au-NP. As demonstrated in [24], the stabilising ligand exerts a significant influence on the sensor’s performance. It has been reported that ligands such as α-methoxypoly (ethylene glycol)-ω-(11-15 mercaptoundecanoate) (PEG–MUA), 3-mercaptopropionic acid (MPA), and citrate demonstrate varying sensor responses to different target gases. Notably, citrate exhibits the highest sensor response (39%) towards 4000 ppm CO_2_. In a study by Lee et al. [25], the size dependency of Au nanoparticles on CuO nanowires (NWs) was investigated. The process of forming the Au-NPs directly on CuO-NWs involved the initial step of depositing Au layers of varying sizes to regulate the diameter of the Au-NPs. This was achieved through the utilisation of a sputtering technique. Subsequent to a heat treatment, the Au layers underwent a conversion process to become Au-NPs by undergoing a rearrangement. The results indicated that a sensor for CO reaches a maximum for Au-NPs with a diameter of 60 nm.

To the best of our knowledge, there are currently no papers available that demonstrate the impact of varying sizes of Au-NPs on the sensor performance of CuO/Cu_2_O chemoresistive-based sensors towards CO_2_. Therefore, the objective of this paper is to investigate the influence of the variation in diameter of Au-NPs on the sensing performance of functionalized CuO/Cu_2_O thin films, fabricated with different techniques, by measuring different target gases.

## 2. Sensor Fabrication

Two distinct methods were employed to produce the gas-sensitive thin films. The first fabrication technique was the thermal evaporation method. The gas-sensitive thin films were structured by photolithography (Mask-Aligner MJB4, Süss MicroTec, Garching, Germany) using a negative photoresist (AZ nLOF 2000, Merck Performance Materials GmbH, Wiesbaden, Germany) on a micro-hotplate chip. The chosen sensing geometry was a circle with a diameter of 450 µm. A Cu layer with a thickness of 50 or 500 ± 5 nm was deposited by the thermal evaporation method (UNIVEX 450, Leybold AG, Cologne, Germany). The thicknesses were selected on the basis that, in contrast to 50 nm, nanowires are growing at 500 nm. A deposition rate of 0.5 ± 0.1 nm/s for Cu at 5.1 × 10^−6^ mbar was employed. After the lift-off in a remover (TechniStrip^®^ NI555, TECHNIC France, La Plaine Saint-Denis, France) for approximately 4 h at 80 °C and rinsing with distilled water, the chip was oxidized on a PZ28-3T hotplate (HP, Harry Gestigkeit GmbH, Düsseldorf, Germany) at 450 ± 5 °C for 4 h, resulting in a mixture of CuO and Cu_2_O. The temperature of the hotplate was monitored using a thermocouple. The initial step in the heating process was to apply heat from room temperature until a target temperature of 450 ± 5 °C was reached. This was achieved by applying a heating rate of 36 K/min. The temperature was then maintained for a duration of 4 h, after which a natural cooling process to room temperature was facilitated, which took approximately 3 h. After that, the samples were carefully removed from the hotplate.

The second fabrication technique was spray pyrolysis, which was carried out using a self-made setup consisting of a PZ28-3T hotplate (HP, Harry Gestigkeit GmbH, Düsseldorf, Germany) connected with a temperature controller (PR5-3T, Harry Gestigkeit GmbH, Düsseldorf, Germany). The gas supply was constructed using PFA flexible tubes and connections (Swagelok Company, Solon, OH, USA) to link the gas flasks with the air atomisation nozzle (Quickmist 1/4QMJ with flat air cap SUQR220B, Spray System Co, Glendale Heights, IL, USA). The pressure system utilised compressed air, a necessity for the control of the opening and closing of the nozzle. Nitrogen (N_2_) was used as the carrier gas, which was connected to the reservoir containing the liquid solution.

Copper (II) acetylacetonate was dissolved in a solution comprising methanol and ethanol in a 1:1 ratio. Methanol is frequently employed as an organic solvent [26]. Nevertheless, the utilisation of alternative solvents or combinations is imperative, contingent on the configuration of the spray pyrolysis system and its constituent components. Nitrogen was employed as the carrier gas to facilitate the formation of a uniform thin film on the chip, which was heated to 450 °C. Subsequently, the sensing layer was structured by photolithography with a positive photoresist (AZ MiR 701, Merck Performance Materials GmbH, Wiesbaden, Germany). Following the etching step in 6 M HCl, a sensing layer with a thickness of 50 nm was produced. Finally, the positive photoresist is removed with a stripper (AZ 100 Remover, Merck Performance Materials GmbH, Wiesbaden, Germany) over a period of 4 h, after which the chip was rinsed with distilled water. After that, the chip was ready for further processing steps.

## 3. Synthesis of Au-Nanoparticles

**Figure 1:**
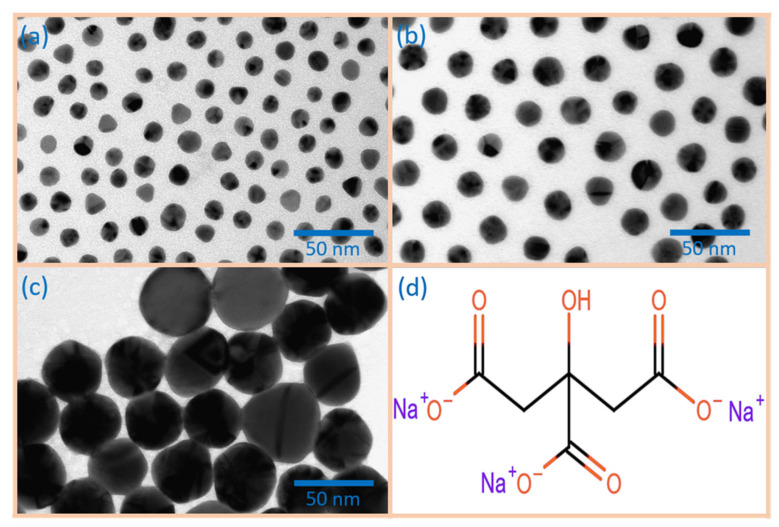
Transmission electron microscopy (TEM) image of (**a**) 12 nm, (**b**) 20 nm, and (**c**) 40 nm Au-NPs stabilized with (**d**) citrate ligands.

Colloidal gold nanoparticles (Au-NPs) with a diameter of 12.2 +/− 1.3 nm, 20.3 +/− 1.1 nm, and 41.2 +/− 4.7 nm (Figure 1a–c) were synthesised for the functionalisation of the metal oxide gas sensors using an inverse Turkevich method [27]. In this wet chemical approach, a citrate buffer, comprising one part citric acid and three parts sodium citrate (Figure 1d), was utilised to regulate the pH. Prior to the synthesis, all glassware was cleaned with aqua regia. In a flask, 1000 mL of 2.5 mM citrate buffer solution was heated to boiling point. The solution was agitated for a period of 10 min at the aforementioned temperature. In parallel, 100 mL of 2.5 mM tetrachloroauric acid was heated to 85 °C and stirred at that temperature. Following a 10-min period of stirring, the solution was rapidly transferred into the boiling buffer solution. The reaction mixture was then cooled to 70 °C and transferred into an appropriate storage container. Prior to the functionalisation of the SMOX gas sensors, the Au-NPs were isolated via centrifugation (15,000× *g* for 25 min) from the reaction mixture and redispersed in high-purity water.

## 4. Sensor Functionalization and Sensor Device

**Figure 2:**
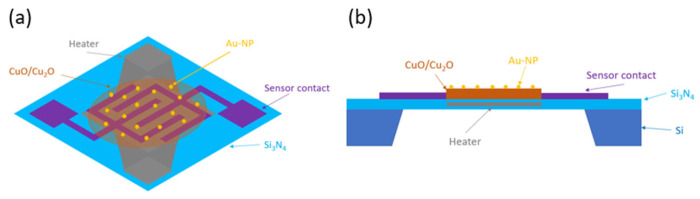
Illustration of a CuO/Cu_2_O gas sensor functionalised with Au-NPs: (**a**) top view [28] and (**b**) side view.

The sensors were functionalised using the same principle as described in reference [28], namely drop coating with citrate or with gold nanoparticles (Au-NPs) of 12, 20, and 40 nm in size. Thus, a drop of 0.2 µL was placed on the sensitive layer and dried for 10 min. This process was repeated twice to ensure sufficient citrate or Au-NPs were applied to the sensitive layer. In Figure 2, the functionalised sensor is displayed in two perspectives: (a) a top view illustrating the electrode structures, and (b) a side view depicting the under-etched Si_3_N_4_ membrane.

The micro hotplate used in this study was fabricated by the former company Applied Sensors (Reutlingen, Germany). Micro-Electro-Mechanical Systems (MEMS) technology was utilised in the fabrication of a 2 × 2 mm^2^ Si_3_N_4_ platform chip. A Si_3_N_4_ membrane, with a thickness of 900 nm and a size of 1.02 × 1.02 mm^2^, was formed by an under-etching process. The sensing structures were fabricated by sputtering technology and contain a heater with Molybdenum (Mo) and Platinum (Pt), along with a Titanium (Ti) adhesion layer as sensor contacts. The heater exhibited a meandering configuration, encompassing an area of 455 × 455 µm^2^. The sensor contacts formed a finger-like structure with a length of 150 µm and a width of 20 µm, covering an area of 340 × 340 µm^2^. The distance between the platinum electrodes was approximately 5 µm. Subsequent to this, the sensing chip was adhered to a “KD-S78382-H CERAMIC DUAL INLINE PACKAGE” (Kyocera Corporation, Kyoto, Japan) by an electrically non-conductive adhesive (LOCTITE ABLESTIK 2035SC, Henkel AG & Co., Düsseldorf, Germany). Au-wires were used to connect the sensing chip to the ceramic package with a wire bonder (53XX BDA, F&S BONDTEC Semiconductor GmbH, Braunau am Inn, Austria). The connected system was then stacked onto a printed circuit board (PCB), which was then ready for measurement in the self-made gas measurement box with a volume of about 80 cm^3^.

## 5. Sensor Characterisation

Raman spectroscopy (WITec alpha 300, WITec Wissenschaftliche Instrumente und Technologie GmbH, Ulm, Germany), using a Zeiss EC Epiplan-Neofluar DIC 100×/0.9 NA objective (Carl Zeiss Microscopy GmbH, Oberkochen, Germany), was employed to ascertain the composition of thermally evaporated and spray pyrolysed unfunctionalized films. In a subsequent study, the distribution of the Au-NPs on the sensing films was characterized by SEM using a Raith eLINE+ instrument (Raith GmbH, Dortmund, Germany).

A self-made gas measurement setup was employed for the characterisation of the gas sensing performance of the sensor devices. The resistance was determined by applying a constant current of 0.1 mA and measuring the voltage. The sensor devices were tested against CO, CO_2_, and HC_Mix_ (a mixture of equal volumes of acetylene, ethane, ethene and propene, with a total concentration of 500 ppm), which were all bought from Linde Gas GmbH, which is headquartered in Dublin, Ireland. The background gas was synthetic air, prepared by Linde Gas GmbH (Dublin, Ireland), with a composition of 80% nitrogen and 20% oxygen. A constant flow of 1000 sccm (equals 1.67 × 10^−5^ m^3^/s) was set by a mass flow controller (EL-FLOW, Bronkhorst High-Tech B.V., Ruurlo, The Netherlands). The dosing of the test gases was achieved by reducing the background gas flow to about the same amount of test gas to keep the gas flow constant at 1000 sccm. CO_2_ concentrations of 500, 1000 and 2000 ppm were applied, and for CO and HC_Mix_, 5, 10 and 20 pp, were selected in accordance with the maximum workplace concentrations. The sensor was heated to an operating temperature of 200 or 300 °C. The relative humidity (r.h.) was set to 50% by a self-made bubbler system and controlled by a commercial humidity sensor (AFK-E, KOBOLD Holding Gesellschaft G.m.b.H., Vienna, Austria). The impact of the size of Au-NPs on the sensor’s response to CO_2_ and other target gases was investigated. The sensor response (S) was calculated using Equation (1), where R_g_ is the highest resistance during the gas exposure and R_a_ is the resistance in synthetic air, which equals the baseline resistance.

(1) $$S=\frac{R_{g}-R_{a}}{R_{a}}*100\%$$

Response time is the duration it takes for the sensor signal to rise from its baseline resistance to 90% of its maximum resistance after gas exposure. Recovery time is the time required for the signal to drop from its maximum resistance to 10% after the gas is removed.

## 6. Results

### 6.1. Raman Investigations

The primitive unit cell of CuO contains four atoms, resulting in 12 distinct vibration modes, as depicted in Equation (2) [29]. The three Raman-active modes are denoted by *A_g_ + 2B_g_*, while the *3A_u_ + 3B_u_* modes are classified as the six infrared-active modes. The three *A_u_ + 2B_u_* modes are classified as acoustic modes.

(2) $$\Gamma_{CuO}=A_{g}+{2B}_{g}+{4A}_{u}+{5B}_{u}$$

Equation (3) illustrates the modes of Cu_2_O [30]. In contrast to CuO, Cu_2_O has only the *T*_2*g*_ mode, which is Raman-active. The *A*_2*u*_ *+ E_u_* are infrared active and the *T*_1*u*_ *+ T*_2*u*_ are silent.

(3) $$\Gamma_{Cu_{2}O}=A_{2u}+E_{u}+{3T}_{1u}+T_{2u}+T_{2g}$$

The group theory results in 42 vibrational modes of Cu_4_O_3_, as outlined in Equation (4) [31]. Of these, 21 (*A*_2*u*_ and *E_u_*) are infrared active, while nine (*A*_1*u*_, *B*_1*u*_ and *B*_2*u*_) are silent. It is important to note that only nine (*A*_1*g*_, *B*_1*g*_ and *B*_2*u*_) are Raman active modes.

(4) $$\Gamma_{Cu_{4}O_{3}}={3E}_{g}+A_{1g}+{2B}_{1g}+{9E}_{u}+{6A}_{2u}+{5B}_{2u}+{2B}_{1u}+{2A}_{1u}$$

**Figure 3:**
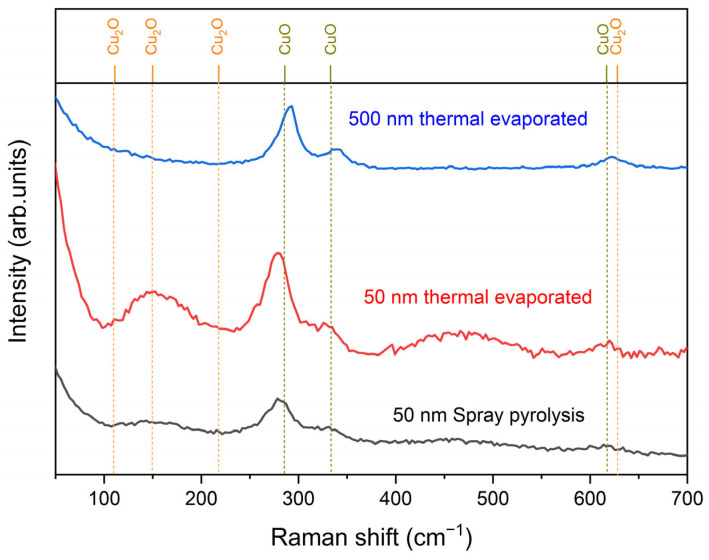
Raman investigations of the three different fabricated CuO/Cu_2_O layers of the sensors.

The outcomes of the Raman investigation are presented in Figure 3. According to the extant literature [32], nanostructured CuO exhibits characteristic peaks at 286, 333, and 617 cm^−1^, as illustrated by the green lines in Figure 3. Conversely, bulk CuO [33] demonstrates higher wavenumbers of 298, 347, and 620 cm^−1^. The influence of grain size on shift has been demonstrated by previous studies [34], which observed wavenumbers of 288, 330, and 621 cm^−1^ for 30 nm grain size, and 295, 342, and 628 cm^−1^ for grain sizes larger than 100 nm. The investigation further demonstrated the presence of CuO in all fabricated sensor films.

In contrast, the Cu_2_O exhibits peaks at the typical wavenumbers 111, 150, 216, and 628 cm^−1^ [29]. In the Raman investigations, the 50 nm thermally evaporated film exhibited a broad peak around 150 cm^−1^, frequently attributed to defects and non-stoichiometry in Cu_2_O [30]. In contrast, spray pyrolysed samples exhibited significantly smaller peaks at 111, 150, and 216 cm^−1^. In addition, Raman investigations on sensors with 500 nm thermally evaporated films did not show any evidence of Cu_2_O.

In the case of Cu_4_O_3_ [30], peaks are denoted at wavenumbers 318, 510, 541, and 651 cm^−1^. However, Raman investigations revealed that Cu_4_O_3_ was not detected in any of the sensors.

### 6.2. SEM Investigations

**Figure 4:**
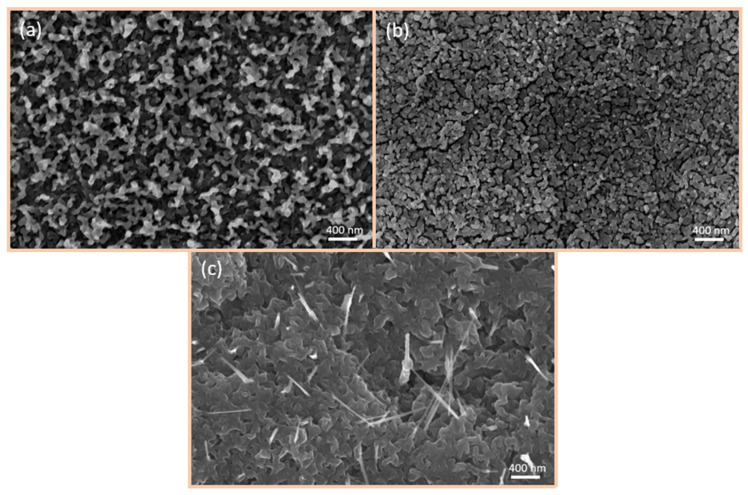
SEM images of three different CuO/Cu_2_O films fabricated using three distinct methods: (**a**) spray pyrolysis of 50 nm, (**b**) thermal evaporation of 50 nm, and (**c**) thermal evaporation of 500 nm.

SEM images of the variously fabricated CuO/Cu_2_O surfaces are presented in Figure 4. The utilisation of spray pyrolysis (Figure 4a) resulted in the formation of a porous, sleet-shaped structure with a considerable surface area. In the case of the 50 nm thermally evaporated CuO/Cu_2_O films (Figure 4b), the surface was observed to be more compact and therefore more densely packed. In contrast, the 500 nm thermally evaporated CuO/Cu_2_O films (Figure 4c) exhibited the highest density and the presence of CuO-nanowires (NWs) on the surface. The growth of CuO-NWs is caused by the oxidation process [35]. Initially, a Cu_2_O layer is formed on the Cu surface. Further oxidation then leads to the formation of a CuO layer on top. Cu ions subsequently diffuse along the grain boundaries through this layer system to the surface, where NW growth then commences. If the NWs are too long, they break down on the surface, which also correlates with our observations.

**Figure 5:**
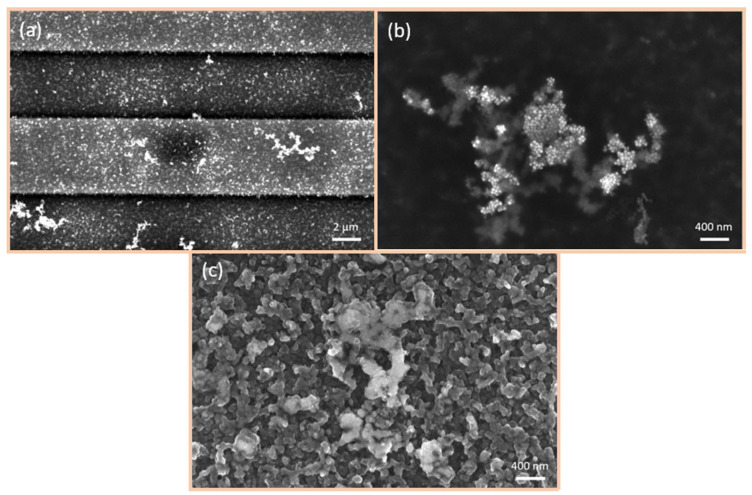
SEM images of CuO/Cu_2_O films fabricated by spray pyrolysis and functionalised with 40 nm Au-NPs: (**a**) overview of the sensor surface after functionalisation with Au-NP, (**b**) Au-NP before heating to 300 °C, and (**c**) Au-NP after heating to 300 °C.

Figure 5 shows SEM images of the surface of the sensors fabricated by spray pyrolysis following functionalisation with 40 nm Au-NPs. The entire surface is covered by Au-NPs, and the formation of larger agglomerations, up to µm in diameter, is also observed (Figure 5a). The Au-NPs are stuck together, yet remain discernible as discrete particles, as evidenced in Figure 5b. Here, it is hard to detect single Au-NPs due to the surface structure, which is porous and sleet-shaped. Upon reaching an operating temperature of 300 °C (Figure 5c), the Au-NPs begin to coalesce, forming larger particles that are no longer separable. However, it is noteworthy that the Au-NPs begin to integrate into the surface. Due to the surface morphology, it is hard to detect separated Au-NPs on the surface.

**Figure 6:**
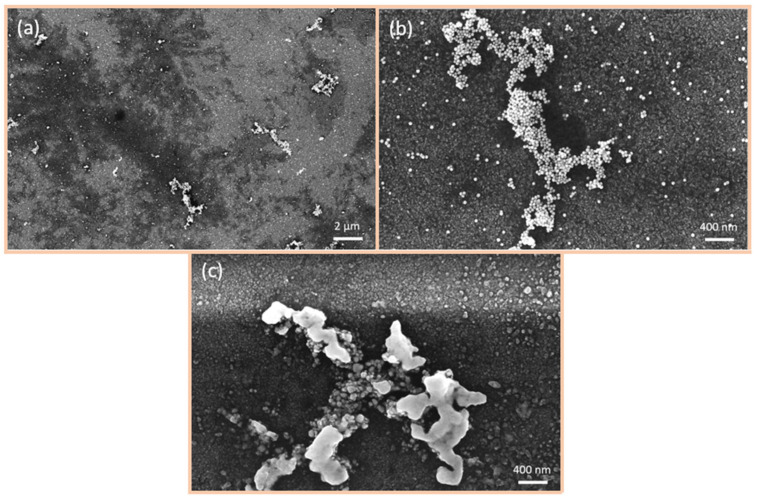
SEM images of 50 nm CuO/Cu_2_O films fabricated by thermal evaporation and functionalised with 40 nm Au-NPs: (**a**) overview of the sensor surface, (**b**) Au-NP before heating to 300 °C, and (**c**) Au-NP after heating to 300 °C.

Figure 6 depicts SEM images of the 50 nm thermally evaporated CuO/Cu_2_O films following functionalisation with 40 nm Au-NPs. The Au-NPs also cover the entire surface of the sensor and form large agglomerates (Figure 6a). As illustrated in Figure 6b, the Au-NPs adhere to one another, yet there are also individual Au-NPs present on the surface. Following the operation of the sensor at 300 °C (Figure 6c), the Au-NPs within the larger agglomerates begin to coalesce, forming larger particles. However, there are still separated Au-NPs observed.

**Figure 7:**
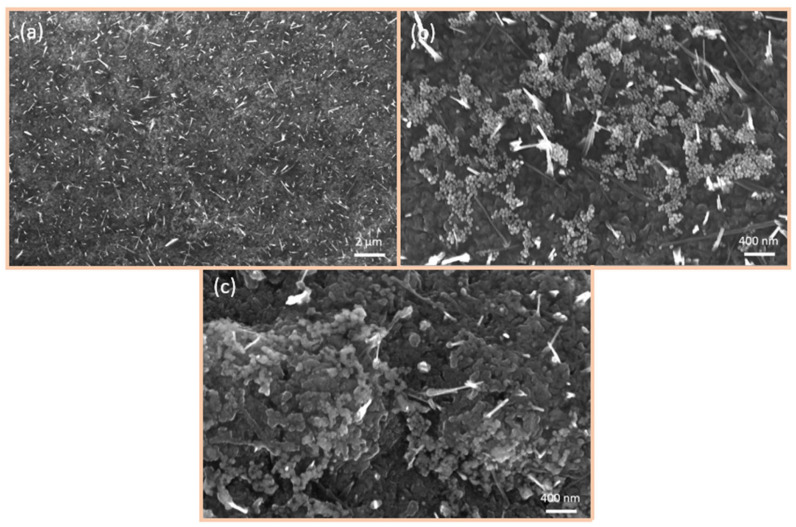
SEM images of 500 nm CuO/Cu_2_O films fabricated by thermal evaporation and functionalised with 40 nm Au-NPs: (**a**) overview of the sensor surface, (**b**) Au-NP before heating to 300 °C, and (**c**) Au-NP after heating to 300 °C.

Figure 7 illustrates SEM images of the 500 nm thermally evaporated CuO functionalised with 40 nm Au-NPs. Figure 7a presents an overview of the sensor surface, wherein the Au-NPs are observed to cover the entire surface, and the formation of larger agglomerates comprising Au-NPs is discerned. Figure 7b illustrates that the Au-NPs remain separated as particles, with individual particles also visible. Adhesion of the Au-NPs to the CuO-NWs is also evident. Following the heating of the sensor, the Au-NPs exhibit significant coalescence, as shown in Figure 7c. This indicates the onset of integration of the Au-NPs into the surface, but there are still individual Au-NPs remaining.

### 6.3. Sensor Performance

This section presents the findings of the gas measurements operated at 300 °C. At 200 °C, neither the pure CuO/Cu_2_O films nor the functionalised variants exhibited any resistance change in response to any of the test gases during an exposure time of 5 min. This indicates that an operating temperature of 300 °C is necessary for the CuO/Cu_2_O films to function. It is evident that elevated temperatures higher than 300 °C can also induce new challenges. This is due to a reduction in sensor response and a decrease in the lifespan of the sensing layer, as shown by Wimmer-Teubenbacher [23].

#### 6.3.1. Resistance Measurements

**Figure 8:**
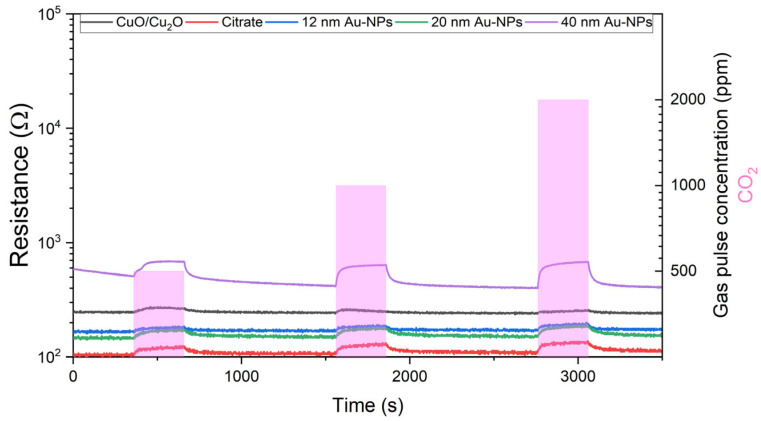
Resistance measurements at 50% r.h. against 500, 1000, and 2000 ppm CO_2_ exposure of spray pyrolysed CuO/Cu_2_O and citrate functionalised samples, as well as samples functionalised with 12, 20, and 40 nm Au NPs.

Figure 8 illustrates the resistance measurements obtained during the exposure of CO_2_ for sensors based on spray pyrolysis. It can be observed that the sensor signal is characterised by a certain degree of noise, with the exception of the sensor that has been functionalised with 40 nm Au-NP. It is evident that all sensors exhibit a change in resistance during exposure to CO_2_ gas. The bare CuO/Cu_2_O sensor exhibits a minimal change in resistance during CO_2_ exposure. The utilisation of 12 nm Au-NPs resulted in a slight increase in the resistance change. In comparison, the resistance change for citrate and 20 nm Au-NPs was similar but considerably higher than that observed for 12 nm Au-NPs. Nevertheless, the highest resistance change for CO_2_ was achieved by the sensor functionalised with 40 nm Au-NPs.

**Figure 9:**
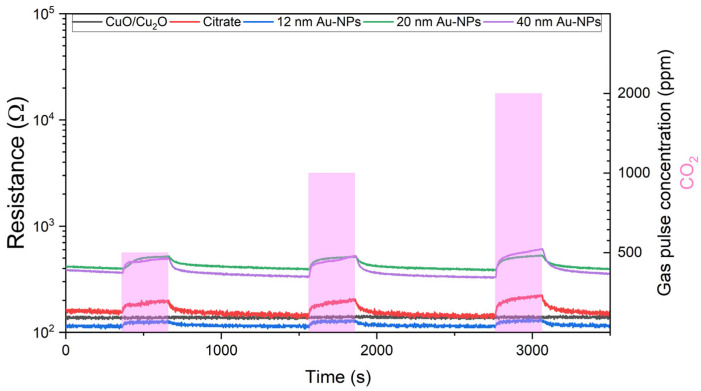
Resistance measurements at 50% r.h. against 500, 1000, and 2000 ppm CO_2_ exposure of 50 nm thermally evaporated CuO/Cu_2_O and citrate functionalised samples, as well as samples functionalised with 12, 20, and 40 nm Au NPs.

Figure 9 shows the outcomes of the 50 nm thermally evaporated CuO/Cu_2_O films in the resistance measurements undertaken during CO_2_ exposure. In comparison to the unfunctionalized spray pyrolysed film, the bare 50 nm thermally evaporated CuO/Cu_2_O film does not demonstrate a change in resistance during CO_2_ exposure. The functionalisation with citrate results in a significant change in resistance during exposure, although the signal remains noisy. In the case of 12 nm Au-NPs, the signal is also noisy, and the resistance change is reduced. The use of 20 nm Au-NPs stabilises the signal and increases the resistance change. The highest resistance change is achieved by the 40 nm Au-NPs, and the signal is also stable.

**Figure 10:**
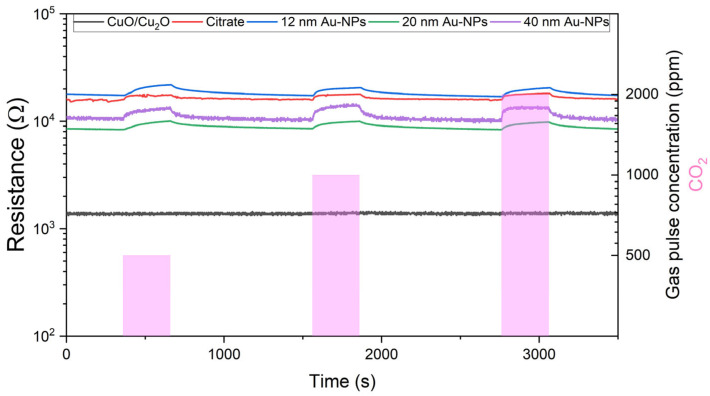
Resistance measurements at 50% r.h. against 500, 1000, and 2000 ppm CO_2_ exposure of 500 nm thermally evaporated CuO/Cu_2_O and citrate functionalised samples, as well as samples functionalised with 12, 20, and 40 nm Au NPs.

Figure 10 illustrates the resistance measurements taken during the exposure of CO_2_ to sensors comprising 500 nm thermally evaporated CuO. In comparison to the 50 nm thermally evaporated sensors and the spray pyrolysed sensors, the baseline resistance of the 500 nm thermally evaporated functionalised sensors is markedly elevated. The pure CuO/Cu_2_O does not exhibit a change in resistance during the exposure period. The sensor signal of the pure CuO/Cu_2_O and the sensors functionalised with 40 nm Au-NPs also exhibited considerable noise. However, the functionalisation of the sensing layer results in an increase in the baseline resistance and a change in the resistance during the exposure to the gas. The resistance change during the exposure is relatively small when citrate is used without Au-NPs. The 12 and 20 nm Au-NPs result in a slight increase in the resistance change during the CO_2_ exposure. The greatest change in resistance is achieved by the 40 nm Au-NPs.

#### 6.3.2. Response and Recovery Time

The response and recovery times for 2000 ppm CO_2_ of the various CuO/Cu_2_O films functionalised with citrate and different sizes of Au-NPs are presented in Table 1. The other gases show the same behaviour. Nevertheless, in this instance, it proves challenging to discern any discernible dependency of the response time on the substrate, nor any discernible influence exerted by the Au-NPs. With regard to the recovery times, it can be posited that the spray pyrolysed CuO/Cu_2_O shows the smallest values, in comparison with the thermal evaporation of CuO/Cu_2_O. The thickness of the thermally evaporated CuO/Cu_2_O is also a contributing factor, as evidenced by the 500 nm data, which demonstrate the longest recovery times.

#### 6.3.3. Sensor Response

- Sensing performance for CO

**Figure 11:**
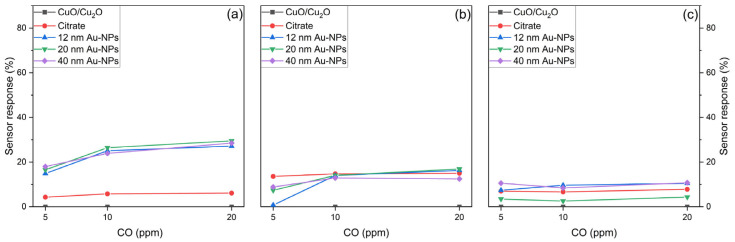
Sensor response of spray pyrolysed (**a**), 50 nm thermally evaporated (**b**), and 500 nm thermally evaporated (**c**) bare CuO/Cu_2_O functionalised with citrate, as well as functionalised with 12, 20, and 40 nm Au NPs against 5, 10, and 20 ppm CO at 50% r.h.

As illustrated in Figure 11, the sensor response towards CO is shown at concentrations of 5, 10, and 20 ppm for all three sensor base layers, along with the functionalisation of citrate and Au-NPs with sizes of 12, 20, and 40 nm, respectively. Figure 11a shows the sensors based on spray pyrolysis; here, the bare CuO/Cu_2_O does not react to the CO, while the citrate-functionalised sensors show a small increase in sensor response. The sensors functionalised with Au-NPs have a higher sensor response compared to the citrate-functionalised sensors, and they reach saturation at concentrations above 10 ppm of CO, as no further increase in sensor response is visible. Consequently, these sensors are only capable of discerning lower concentrations of CO. It is noteworthy that the dimensions of the Au-NPs do not appear to exert an influence on the sensor response of the spray pyrolysed CuO/Cu_2_O films, as all of them exhibit a comparable sensor response towards CO.

In the case of the 50 nm thermally evaporated sensors (Figure 11b), a saturation point of 10 ppm is also achieved for all sensors that were functionalized. It is evident that the sensor with citrate functionalisation exhibits a sensor response that is analogous to that of the Au-NP functionalised sensors. The bare CuO/Cu_2_O also does not show a response to CO.

Finally, the 500 nm thermally evaporated sensors (Figure 11c) demonstrate an inability to differentiate between varying concentrations of CO, as the sensor responses of each functionalisation method are comparable, except for the bare CuO/Cu_2_O, which does not show any sensor response.

- Sensing performance for CO_2_

**Figure 12:**
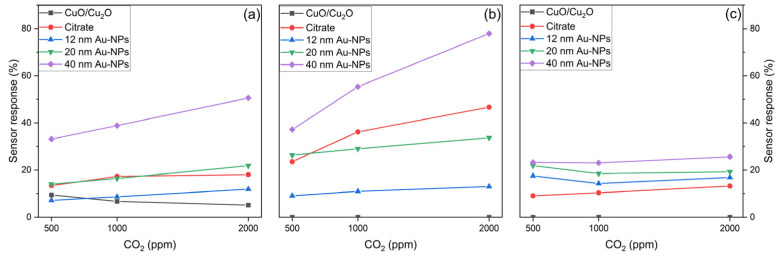
Sensor response of spray pyrolysed (**a**), 50 nm thermally evaporated (**b**), and 500 nm thermally evaporated (**c**) bare CuO/Cu_2_O functionalised with citrate, as well as functionalised with 12, 20, and 40 nm Au NPs against 500, 1000, and 2000 ppm CO_2_ at 50% r.h.

In Figure 12 the sensor responses of the sensing layers to CO_2_ are presented. In the case of spray pyrolysed sensors (Figure 12a), the bare CuO/Cu_2_O demonstrates a reduction in sensor response with increasing CO_2_ concentration, indicative of inadequate sensor recovery. Conversely, citrate-functionalised sensors attain saturation at concentrations of 1000 ppm. The functionalisation with Au-NPs, on the other hand, results in a concentration-dependent sensor response, with an increase in sensor response with increasing CO_2_ concentrations. The highest sensor response is achieved by the 40 nm Au-NPs.

The 50 nm thermally evaporated sensors (Figure 12b) demonstrate a comparable behaviour, with Au-NPs and citrate-functionalised sensors exhibiting an increase in sensor response with rising CO_2_ concentrations. Conversely, the bare CuO/Cu_2_O does not exhibit a sensor response to CO_2_. The sensor response exhibited a moderate increase in the case of the 12 and 20 nm Au-NPs, as the CO_2_ concentration increased. However, the citrate and 40 nm Au-NPs demonstrated a more pronounced increase in the sensor response, with the 40 nm Au-NPs exhibiting the highest sensor response.

In the case of the 500 nm thermally evaporated sensor (Figure 12c), the bare CuO/Cu_2_O also does not show a response towards CO_2_. However, the functionalisation with Au-NPs or citrate leads to a sensor response, but these functionalised sensors are unable to distinguish between different concentrations, as the sensor response does not change significantly. It is noteworthy that the sensor response is reduced for 12 and 20 nm Au-NPs at concentrations of 1000 ppm and 2000 ppm, respectively, in comparison to the sensor response at a concentration of 500 ppm.

- Sensing performance for HC_Mix_

**Figure 13:**
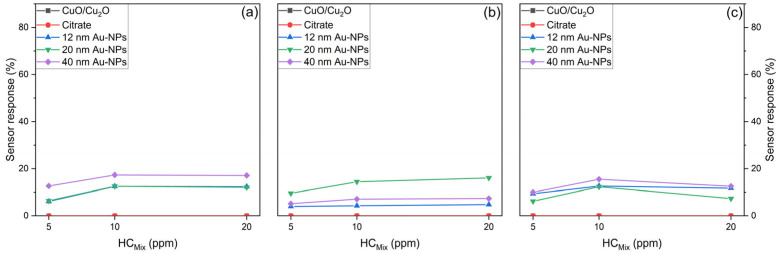
Sensor response of spray pyrolysed (**a**), 50 nm thermally evaporated (**b**), and 500 nm thermally evaporated (**c**) bare CuO/Cu_2_O functionalised with citrate, as well as functionalised with 12, 20, and 40 nm Au NPs against 5, 10, and 20 ppm HC_Mix_ at 50% r.h.

Figure 13 shows the sensor response of various sensors to HC_Mix_. It is evident that the presence of Au-NPs on the sensors is a prerequisite for the measurement of the sensor response to HC_Mix_. In contrast, the bare CuO/Cu_2_O and the citrate-functionalised sensor exhibited no measurable response to HC_Mix_. In the case of Au-NPs, the spray pyrolysed sensors (Figure 13a) attain a saturation level of sensor response at 10 ppm of the HC_Mix_, as no further increase of the sensor response is detected for 20 ppm. It is evident that the highest sensor response is achieved by 40 nm Au-NPs, while 12 and 20 nm Au-NPs show a similar sensor response towards the HC_Mix_. The 50 nm thermally evaporated sensors (Figure 13b) demonstrate a moderate increase for the 20 nm Au-NPs, while the sensors functionalised with 12 and 40 nm Au-NPs are saturated at 10 ppm. In this case, the 12 nm Au-NPs yield the most significant sensor response towards HC_Mix_. Conversely, the 500 nm thermally evaporated sensors (Figure 13c) functionalised with Au-NPs exhibited a decline in sensor response at 20 ppm compared to the 10 ppm, suggesting a recovery issue for the sensors due to the high sensor saturation. It is notable that the sensor response of all Au-NPs is similar.

#### 6.3.4. Comparison of Sensor Response

**Figure 14:**
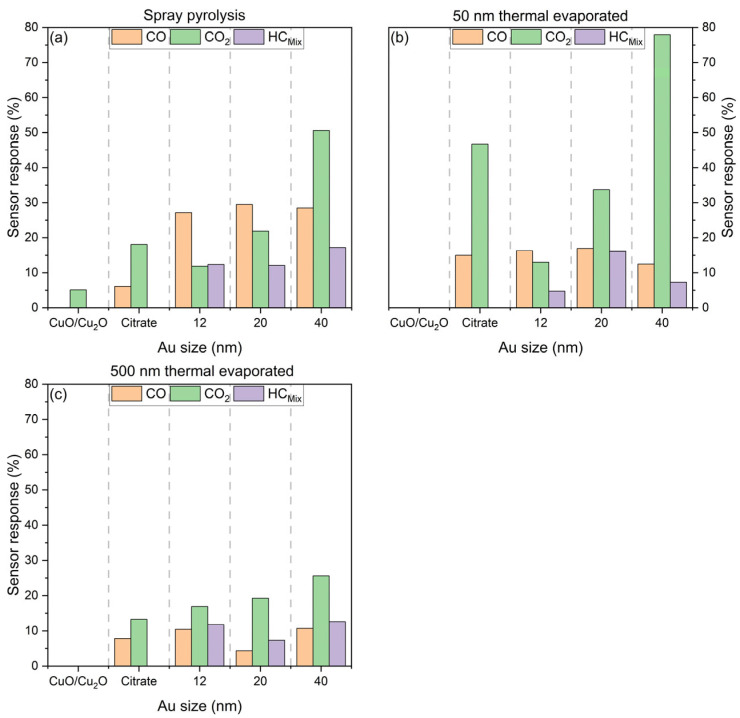
Sensor response of the three fabrication methods: (**a**) spray pyrolysis, (**b**) 50 nm thermally evaporated, and (**c**) 500 nm thermally evaporated CuO/Cu_2_O and citrate-functionalised samples, as well as samples functionalised with 12, 20, and 40 nm Au NPs towards 2000 ppm CO_2_, 20 ppm CO, and HC_Mix_ at 300 °C and 50% r.h.

Figure 14 provides a summary of the sensor response (calculated with Equation (1)) for all three sensor layers when exposed to the highest concentrations of three test gases, namely CO (20 ppm), CO_2_ (2000 ppm), and HC_Mix_ (20 ppm).

The sensor responses of the spray pyrolysed sensors are illustrated in Figure 14a. The sensors comprising pure CuO/Cu_2_O produced via the spray pyrolysis method exhibited a relatively low sensor response of 5.1% to CO_2_. Functionalisation with citrate enabled a low sensor response of 6.1% towards CO and increased the sensor response of CO_2_ to 18%. However, the utilisation of disparate Au-NP sizes resulted in a sensor response between 27% and 29.5%. In the case of CO_2,_ the sensor response is reduced to 11.9% for 12 nm Au-NPs in comparison to the citrate without Au-NPs. An increase in the size of the Au-NPs to 20 nm also resulted in an enhanced sensor response of 21.8%. Nevertheless, the highest sensor response of 50.5% was observed with the 40 nm Au-NPs for CO_2_. Conversely, the use of Au-NPs is essential for the detection of HC_Mix_, as neither the pure CuO/Cu_2_O nor the citrate-functionalised sensors exhibited any response. The sensor response with Au-NPs is observed to be between 12% and 17.1% for the HC_Mix_.

Figure 14b illustrates the sensor response of the 50 nm thermally evaporated films to different test gases. It is evident that the pure CuO/Cu_2_O film exhibits no reaction to any of the test gases. However, functionalisation with citrate or Au-NPs facilitates the detection of CO, resulting in a sensor response ranging from 12.4 to 16.9%. In the case of CO_2_, the citrate-functionalised sensor achieves a sensor response of 46.7%, while the Au-NPs with 12 and 20 nm diameters only result in a lower sensor response of 13% and 33.7%, respectively. The highest sensor response of 78% is achieved by the 40 nm Au-NPs for CO_2_. The behaviour exhibited by the HC_Mix_ is analogous to that observed in the case of the spray pyrolysed sensors, with the utilisation of Au-NPs resulting in a sensor response. In this instance, the employment of 20 nm Au-NPs attains the maximum sensor response of 16.1% for the HC_Mix_, while the use of 12 and 40 nm Au-NPs yields a substantially lower sensor response of 4.7% and 7.3%, respectively.

The sensor responses for the 500 nm thermally evaporated films are illustrated in Figure 14c, and a similar behaviour is observed for the 50 nm thermally evaporated films. Pure CuO/Cu_2_O does not show a sensor response to any of the test gases, and the functionalisation with citrate or Au-NPs enables a sensor response in the range of 4.3 and 10.7% to CO. In the case of CO_2_, functionalisation with citrate results in the lowest sensor response of 13.2%. However, for Au-NPs, an increase in size leads to an increase in sensor response. The highest sensor response of 25.6% for CO_2_ is achieved using 40 nm Au-NPs. In direct comparison, the sensor response for CO_2_ is significantly lower than that of the 50 nm thermally evaporated sensors. The sensor response of HC_Mix_ displays a similar trend to that of the spray pyrolysed and 50 nm thermally evaporated sensors, and the Au-NPs are also necessary to achieve a sensor response within the range of 7.3 and 12.6%.

In terms of selectivity, the results indicate a high degree of potential for the development of a gas sensor array comprising bare CuO/Cu_2_O fabricated by spray pyrolysis, citrate on one of the three CuO/Cu_2_O, as well as the 12 nm and 40 nm Au-NPs on the spray pyrolysed CuO/Cu_2_O. For instance, if all sensors react to a gas mixture, this strongly indicates the presence of a CO_2_ component. Conversely, if only the citrate-functionalised sensors react, CO is measured. However, if only the 12 or 40 nm Au-NPs sensor reacts, this indicates the presence of HC_Mix_. A comparison of the responses of the 12 nm and 40 nm Au-NPs to CO reveals that the former exhibits a more pronounced response, while the latter demonstrates a significantly higher response to CO_2_.

## 7. Summary and Discussion

Table 2 shows a comparison with other chemoresistive CuO-based gas sensors for the detection of CO_2_.

In this study, we have successfully fabricated a metal oxide gas sensor on a Si_3_N_4_ micro hotplate (µh) chip with Pt electrodes and Mo heaters for chemoresistive gas measurements. The influence of varying Au-NP sizes (12, 20, and 40 nm, verified by TEM) and of the pure citrate on the sensor performance has been investigated. The composition of the sensing layer was confirmed through Raman spectroscopy, where the spray pyrolysed and thermally evaporated layers consist of CuO/Cu_2_O, and the 500 nm thermally evaporated layer consists of CuO only. The surface structure was analysed using SEM, which showed that the surface strongly depends on the method and thickness used, with all three sensing layers being different. The surface fabricated by spray pyrolysis was found to exhibit a porous structure and the shape of sleet. In comparison, the surface that was fabricated with 50 nm thermal evaporation was found to be more compact and thus more densely packed. The surface fabricated by 500 nm thermal evaporation was found to demonstrate the highest density, including CuO-NWs on the surface. Furthermore, observations made using SEM revealed that Au-NPs tend to agglomerate on the surface prior to operating the sensor device. This is followed by coalescence at the operating temperature of 300 °C, a phenomenon that has been documented in the literature, where small NPs at high temperatures are not stable [39].

Gas measurements were performed on the pure and functionalised films for CO_2_, CO, and HC_Mix_ to assess the cross-sensitivity at 50% r.h. Initially, the functionalisation with Au-NPs stabilises the baseline resistance in comparison to the noisy signal of the pure CuO/Cu_2_O. It has been demonstrated that, in the case of certain functionalised sensors, there is a shift in the baseline resistance towards higher values in comparison with the bare CuO/Cu_2_O. The investigation revealed that the pure CuO/Cu_2_O, fabricated by spray pyrolysis, exhibited a sensor response to CO_2_, in contrast to the films produced by thermal evaporation.

In general, SMOX sensors demonstrate minimal or no response to CO_2_. However, as outlined in reference [23], an effective CO_2_ sensor based on CuO is presented, accompanied by a proposed sensing mechanism. A comprehensive explanation of the sensing mechanism of CO_2_ by CuO remains to be fully elucidated. Furthermore, the utilisation of CuO in combination with other materials for the detection of CO_2_ is described in the review paper by Maier et al. [9].

The functionalization of citrate was found to enhance the sensor response to CO_2_ and enable a sensor response to CO. Furthermore, it was observed that functionalisation with Au-NPs resulted in a sensor response for HC_Mix_ and enhanced the sensor response towards CO_2_. However, it was also demonstrated that a concentration dependency is exhibited exclusively for CO_2_. The majority of the functionalised sensors attain a saturation level at 10 ppm of CO or HC_Mix_, thereby precluding the discernment of higher concentrations. The size of the Au-NPs has been shown to have a significant impact on the detection of CO_2_. The 40 nm Au-NPs demonstrated the most significant sensor response for all the fabricated sensing layers, with the 50 nm thermally evaporated CuO/Cu_2_O layers exhibiting the highest sensor response of 78%. In general [40], it has been established that a reduction in film thickness results in an enhancement of the sensor response, as surface effects become more pronounced.

In terms of selectivity, the results indicate a high degree of potential for the development of a gas sensor array comprising the following elements: bare CuO/Cu_2_O fabricated by spray pyrolysis; citrate on one of the three CuO/Cu_2_O; and 12 nm and 40 nm Au-NPs on the spray pyrolysed CuO/Cu_2_O. However, further research is required, including the use of other NPs (e.g., Pt, Pd) and alternative fabrication methods such as magnetron sputtering, to generate more comprehensive insights. In addition, the integration of NWs with NPs could prove to be a fruitful avenue for future research.

## Figures and Tables

**Table 1:** Response time of three different CuO/Cu_2_O films fabricated using three distinct methods: spray pyrolysis of 50 nm, thermal evaporation of 50 nm, and thermal evaporation of 500 nm at 2000 ppm CO_2_.

Response Time [s]				
**Method**	**Citrate**	**12 nm Au-NPs**	**20 nm Au-NPs**	**40 nm Au-NPs**
Spray pyrolysis of 50 nm	32.4	70.2	62.1	66.7
Thermal evaporation of 50 nm	87.5	37.3	99.8	107.9
Thermal evaporation of 500 nm	49.5	146.7	125.9	40.6
**Recovery Time [s]**				
**Method**	**Citrate**	**12 nm Au-NPs**	**20 nm Au-NPs**	**40 nm Au-NPs**
Spray pyrolysis of 50 nm	96.9	38.5	84.1	61.5
Thermal evaporation of 50 nm	128.8	65.9	133.3	129.7
Thermal evaporation of 500 nm	137.4	199.5	184.2	135.31

**Table 2:** Comparison to other chemoresistive CuO-based gas sensors for CO_2_.

Morphology	Method	Operating Temperature (°C)	Relative Humidity (%)	CO_2_ (ppm)	Sensor Response	References
CuO/CuFe_2_O_4_ Thick film	Co-precipitation (paste)	350	0	5000	10%	[36]
SnO_2_/CuO with 0.5 wt% Ag Nanospheres	Hydrothermal process	300	Not defined	10,000	72%	[37]
CuO-NPs with ZnO	Drop coating	300	30	1000	12%	[38]
CuO with Au	Drop coating	300	50	2000	78%	This work

## Data Availability

Data are contained within the article.
